# Multi-Serial Adaptive Bus Interface with Integrated Monitoring and Plug-And-Play Connectivity

**DOI:** 10.3390/s25247638

**Published:** 2025-12-16

**Authors:** Marcel Tresanchez, Tomàs Pallejà

**Affiliations:** Department of Industrial and Building Engineering, University of Lleida, 25001 Lleida, Spain; tomas.palleja@udl.cat

**Keywords:** multi-serial interface, bus auto-detection, baud rate supervision, RS-485 direction control, CAN, plug-and-play connectivity, modular hardware, industrial automation

## Abstract

This work presents a complete multi-serial adaptive bus interface system compatible with the most widely used industrial serial communications standards: RS-232, RS-485, RS-422, and CAN. The proposed system automatically detects the connected serial interface type through analog line sensors and dynamically redirects the bus to the appropriate transceiver using a logical multiplexer. This approach aims to simplify the configuration of heterogeneous serial devices in complex and modular integration scenarios, such as body builders in industrial or vehicular systems. The hardware is designed as a scalable PCIe card-based device, allowing multiple adaptive bus interfaces to be integrated within a rack-based modular architecture. In addition, a single 5-pin plug-and-play connector is proposed by unifying the different bus signals of the transceivers, thereby simplifying cabling and deployment. Complementary implemented capabilities include baud rate auto-detection and supervision, as well as automatic direction-control functionality for RS-485 communication. Experimental validation demonstrated that the proposed system successfully detected and redirected all supported interfaces, achieving reliable connection and disconnection within an average time of 2.5 s. Furthermore, the integrated baud rate auto-detection algorithm accurately identified transmission speeds up to 1 Mbps in under 80 ms, while the automatic direction-control capability operated reliably at speeds up to 576,000 bps.

## 1. Introduction

Over the years, serial wired communication interfaces for data transmission have evolved to improve robustness, throughput, and transmission range. One of the earliest industrial standards was RS-232, established in 1960 [1]. It was widely adopted in the following decades to support advancements in computing [2] and, together with the release of the Modbus protocol in 1979, became foundational in industrial automation systems [3]. In 1975, RS-422 was introduced as an improvement over RS-232, offering differential signaling with twisted pairs for both transmission and reception [4], which enabled higher speeds, better noise immunity, and longer cable lengths. Subsequently, RS-485 was released in 1983, introducing a robust multipoint topology with balanced signaling that supported multiple nodes over a single bus [5]. A similar approach was adopted in 1986 by Bosch GmbH, leading to the development of the CAN (Controller Area Network) standard. CAN introduced a message-based protocol with broadcast communication, data integrity, and prioritization mechanisms, which facilitated widespread adoption in automotive and industrial applications [6,7].

Although Ethernet-based protocols currently dominate industrial automation networks, and the USB (Universal Serial Bus), introduced in 1996, has become the de facto standard for computer and peripheral connectivity, many actual devices and subsystems in large-scale automation systems still rely on legacy serial interfaces such as RS-232, RS-485, RS-422, and CAN. For instance, ref. [8] employs RS-232 and RS-485 for the motion monitoring and control of a 6DoF robotic platform. In [9], RS-485 enables communication with intermediate nodes for low-resource IoT devices. RS-232 and RS-485 are used in [10] as a low-cost communication solution for a remotely operated underwater vehicle. In [11], machine learning techniques are proposed to detect attacks on Modbus over RS-485 links. RS-422 is utilized in [12] to connect the flight control computer and telemetry with the control system of a hybrid vehicle. CAN-based networks are used in [13] for climate monitoring in agricultural systems and in [14] to integrate sensors, actuators, and controllers in mobile robotics.

One of the major challenges in multi-protocol industrial automation systems lies in the wiring and configuration of the various communication interfaces involved. While Ethernet and USB technologies benefit from standardized networking and plug-and-play capabilities through switches and hubs, legacy serial interfaces typically operate independently, lacking universal automatic configuration mechanisms. Approaches in [15,16] have attempted to address this issue by introducing multi-serial interfaces that consolidate several serial standards into a simplified port. Although these solutions are conference contributions, they remain the closest and most recent studies available that address this problem. These solutions aim to reduce the number of independent serial ports required in a centralized processing unit, facilitate manual cabling and configuration tasks, and provide a more flexible setup while maintaining the same number of ports.

A multi-serial-to-Ethernet bridge using a multiprotocol transceiver is implemented in [15]. This solution simplifies the integration of different serial interfaces into an Ethernet network by using the same hardware for RS-232, RS-485, and RS-422. While useful in systems without native serial support, it still requires manual interface selection and does not address the control of multiple RS-485 direction lines. A more advanced system was presented in [16], introducing a multi-serial bus with adaptive detection based on monitoring bus voltage levels. Their proposal defines a simplified external 5-pin connector supporting RS-232, RS-485, RS-422, and CAN. A level-processing unit combined with an ADC integrated circuit supplies voltage information to the main processor, which identifies the interface type and redirects the bus using mechanical relays. Despite simplifying hardware resources, wiring, and configuration complexity in multi-serial environments, this architecture exhibits several potential limitations:

- It performs protocol conversion to a fixed RS-232 interface for communication with the PC, which limits flexibility in scalable architectures.
- The main processor simultaneously performs bus data transmission and bus monitoring, potentially limiting transmission speed and hindering detection of device disconnection during communication.
- Interface switching relies on a complex mechanical relay arrangement to ensure electrical isolation.
- Additional circuitry is required to adapt the bus voltage levels to the ADC’s valid operating range.

In this work, an improved multi-serial adaptive bus interface system with integrated monitoring and universal plug-and-play (PnP) connectivity is presented. The proposed solution is based on a modular rack-mounted architecture in which the dedicated interface processor is exclusively responsible for controlling and monitoring the bus, rather than acting as a protocol bridge. The physical bus connection is established directly between the external serial interface and the upper-layer communication unit. Thus, the system is dedicated to monitoring electrical variations on the bus, selecting the appropriate transceiver, and detecting TTL signal edges required for the additional integrated functionalities.

The main contributions of this work are as follows:

- A hardware architecture that unifies RS-232, RS-485, RS-422, and CAN signals into a simplified 5-pin bus interface, maintaining compatibility while eliminating mechanical relays.
- A sensing strategy that uses the bus protection components to monitor the communication lines through a non-intrusive, unaltered sensing approach.
- A multi-serial switching method performed at the TTL level using a single logical multiplexer, reducing hardware complexity and requiring only one shared TTL interface on the upper-layer processor.
- The definition and implementation of an automatic direction-control mechanism for half-duplex RS-485 communication, integrated into the same processor.
- The development of a baud-rate detection mechanism with real-time anomaly reporting, including mismatch and deviation detection, running on the same processor.

**Figure 1:**
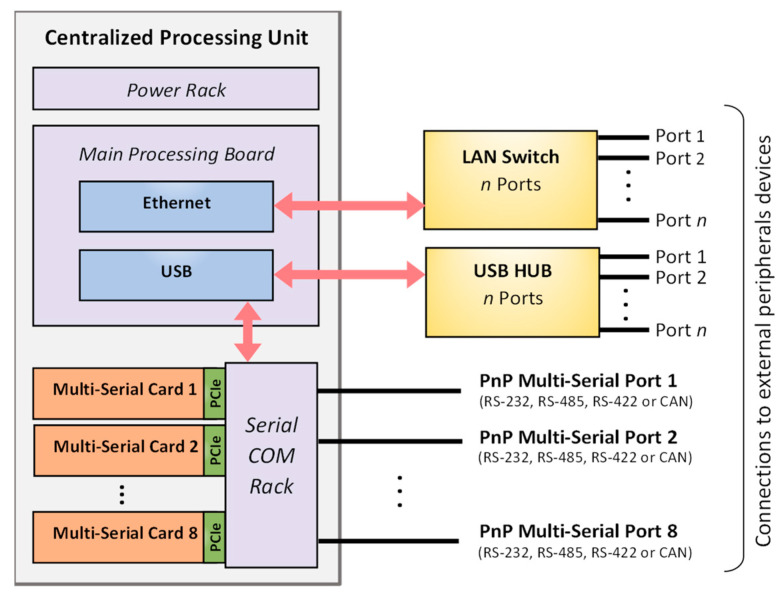
Connection diagram of a centralized processing unit in a smart industrial system using the proposed multi-serial bus interface card.

The hardware of the multi-serial adaptive bus interface is implemented as a flexible and scalable architecture using a compact PCIe expansion card designed for modular integration into rack-based systems. This allows multiple multi-serial adaptive interface cards to be installed in the central unit of an industrial automation controller (Figure 1). The solution is especially suited to industrial platforms requiring flexible integration of modular aftermarket peripherals, such as the onboard systems of industrial fleet vehicles [17,18,19]. This work focuses on the hardware and software implementation of the multi-serial adaptive bus card and does not address how the serial rack concentrator and the central processor manage multiple simultaneous ports.

## 2. Preliminary Study

### 2.1. RS-232

RS-232 is a standard for serial binary data communication between terminal equipment and circuit-terminating equipment standardized by the Telecommunications Industry Association, TIA-232-F [20]. It is an unbalanced, point-to-point, full-duplex asynchronous communication that uses special data bits to identify the frame (start and stop bits) and improve the data integrity (parity bit). RS-232 was originally designed to communicate between a modem and a computer through DB25, and later DB9 serial ports, with distances no more than 15 m at maximum speeds of 19,200 bps. Nowadays, it is used for communication between computers or between a computer and an external peripheral with transceivers capable of reaching up to 1 Mbps.

**Figure 2:**
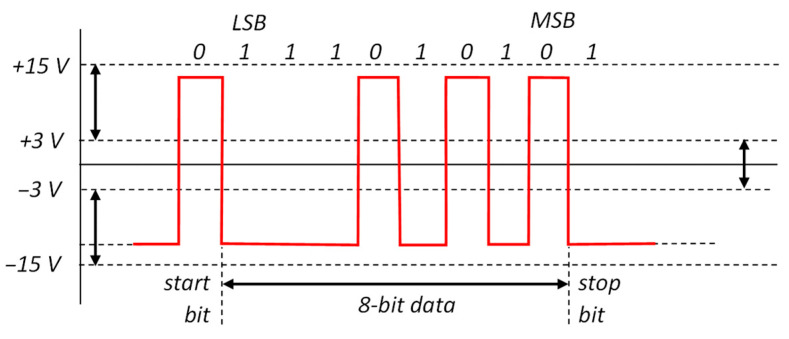
Example of RS-232 transmission of the “W” ASCII character.

RS-232 has one digital signal for each asynchronous receiving and transmitting direction, both referenced to ground. The digital signal is bipolar, commonly between +10 V and −10 V, and, according to the specification, within a limit of ±25 V and a transition threshold of ±3 V. Figure 2 shows an example of a transmitted signal corresponding to the “W” ASCII char (8 bits with binary code 01010111), with no parity bit and one bit of stop. The logic 1 (called mark) is represented as a negative voltage, whereas the logic 0 (called space) is signaled by a positive voltage.

In this work, the Maxim Integrated MAX3227ECAE+T [21] RS-232 transceiver is selected to implement the RS-232 serial communication bus. This transceiver supports 3 V TTL communication at speeds up to 1 Mbps and includes an external pin to disable the output driver, which is a key feature when the output must be shared with other transceivers. Communication tests show that the output voltage ranges between +5.5 and +5.8 V for logic 0 and between −5.6 and −5.7 V for logic 1. When communication is idle, the signals remain negative (logic 1) with the TX line held at the voltage level provided by the connected transceiver. In the event of cable disconnection, the RX signal line drops to reference voltage, 0 V.

### 2.2. RS-485

Unlike RS-232, RS-485 communication uses a balanced signal pair for both receiving and transmitting (half-duplex). These two differential lines, called A and B, ensure equal impedances to ground along their lengths, no ground dependence, and reject the common-mode noise and interference when fed to differential devices. RS-485 interface standard, designated TIA-485-A [22] by the Telecommunications Industry Association, specifies the exchange of binary data signals in multi-point, point-to-point, or multi-dropped interconnection devices. The RS-485 standard improves upon RS-232 in terms of distance (up to 1200 m), speed (10 Mbps over 12 m), and network topologies (up to 32 stations).

**Figure 3:**
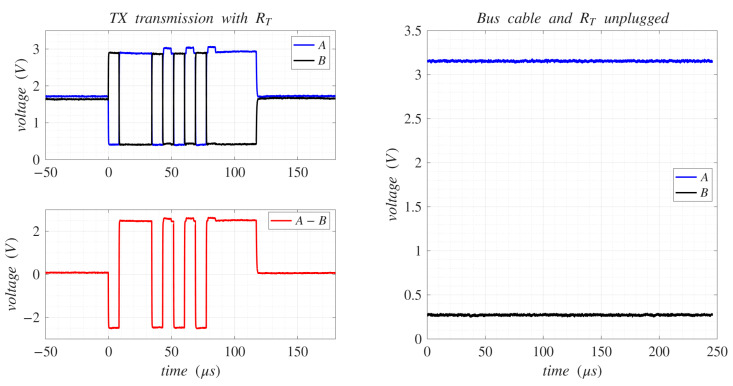
RS-485 transmission of the “W” ASCII character using the SP3485EN-L transceiver (**left**). RS-485 signal status when the twisted pair cable with $R_{T}$ is unplugged (**right**).

The RS-485 standard specifies the bipolar A-B signal with a transition threshold of ±0.2 V and low and high voltage limits of −7 V and +12 V, respectively. Logic 0 is represented by a negative differential voltage, whereas logic 1 is represented by a positive one. Like RS-232, RS-485 typically transmits asynchronous ASCII data frames under the Universal Asynchronous Receiver-Transmitter (UART) peripheral. Figure 3(left) shows the A and B signals referenced to ground, along with the resulting A-B differential signal when transmitting an 8-bit “W” ASCII character at 115,200 bps, with no parity and one stop bit, using the 3.3 V low-power SP3485EN-L [23] transceiver from MaxLinear (Carlsbad, CA, USA). In this configuration, signal A reaches up to 3 V and signal B drops to 0.5 V for a logic 1, and the opposite for a logic 0. The resulting A-B differential voltage is +2.5 V for logic 1 and −2.5 V for logic 0, remaining within the specified limits and transition threshold defined by the standard.

In the transmission example shown in Figure 3(left), a 120 Ω termination resistor ($R_{T}$) was connected across A and B signal wires. A proper termination resistor, equal to the cable impedance (typically 120 Ω at each end of the twisted-pair cable), is essential to prevent signal reflections caused by fast transitions, which can lead to data corruption. It also helps to reduce electrical noise sensitivity due to the lower overall impedance. Figure 3(right), shows the A and B voltage levels when the cable with the $R_{T}$ is unplugged. As shown, the A and B signals shift from their idle levels, both at 1.8 V (Figure 3(left)), to 3.1 V and 0.3 V, respectively. These voltage variations are essential for enabling the automatic detection of the RS-485 physical connection in the proposed multi-serial adaptive system.

**Figure 4:**
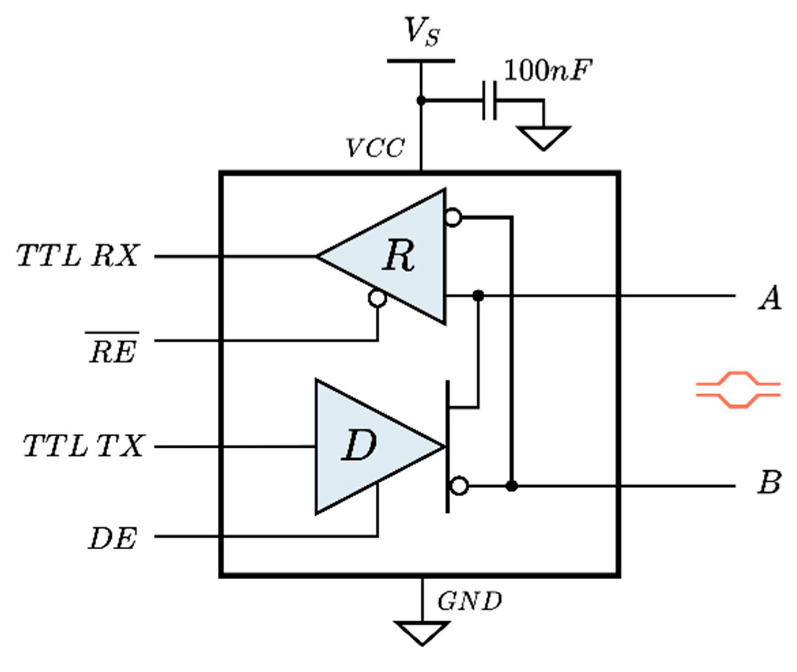
Internal diagram of the SP3485 RS-485 transceiver.

RS-485 transceivers use two tri-state buffers to enable half-duplex communication (Figure 4). Each buffer can be enabled or disabled using external control lines called DE (Drive Enable) for transmission and RE (Receiver Enable) for reception. These digital control lines are managed by the user protocol layer according to the arbitration algorithm that defines when a node is allowed to transmit or receive data. The general usage is to keep the transceiver in receive mode (DE: low, $\bar{RE}$: low) and only switch to transmit mode (DE: high, $\bar{RE}$: high) when data needs to be sent. The capability to fully disable the transceiver (DE: low, $\bar{RE}$: high) is especially useful for saving power but also for keeping the bus free when it is shared with other transceivers, as required in this work.

In the industrial field, balanced signal-pair communication networks are highly exposed to harsh electrical transient events caused by electrostatic discharges, relay contact bounces, inductive loads, or other electrical disturbances. Therefore, compliance with electromagnetic compatibility (EMC) protection standards is required. Level 4 of the IEC 61000-4 transient immunity standard [24], which includes protections for ESD (Electrostatic Discharge, IEC 61000-4-4), EFT (Electrical Fast Transient, IEC 61000-4-4) immunity, and surge events, is recommended. Some transceivers already integrate electrical protection features on the same chip, such as the ADM3095E from Analog Devices (Wilmington, MA, USA). However, in this work, these external protection components are also used to monitor the status of the communication lines, thus avoiding the use of intrusive electronic components on the bus. The set of electronic elements used as electrical protection is as follows:

- **PUL** (Pull-up/down Resistors): Resistors that ensure the transceiver output does not generate logical noise when the lines are not being actively driven by any device (e.g., during startup or plug-in/out events). Common values are 4.7 KΩ or 10 KΩ.
- **TVS** (Transient Voltage Suppressor): Eliminates transient overvoltages on differential lines (e.g., ESDCAN02-2BWY TVS diodes from STMicroelectronics, Geneva, Switzerland).
- **TBU** (Transient Blocking Unit): Protects the TVS by limiting the line current during EFTs. Typical resistance values are around 33 Ω. This element can be replaced by a PTC with equivalent resistance.
- **TISP** (Totally Integrated Surge Protection): Acts as the primary surge protector. When the voltage exceeds a threshold, it diverts most of the transient energy away from the sensitive components. It is typically used as an alternative to TVS and consists of three protection elements between lines (e.g., SMAJ12CA ESD/TVS diodes from Bourns, Riverside, CA, USA).
- **PTC** (Positive Temperature Coefficient Fuse): Self-resetting fuses that disconnect the line during current surges. They prevent major problems such as the burning of TBU resistors (e.g., MF-MSMF010 PTC fuse from Bourns, with 0.3 A trip current).

**Figure 5:**
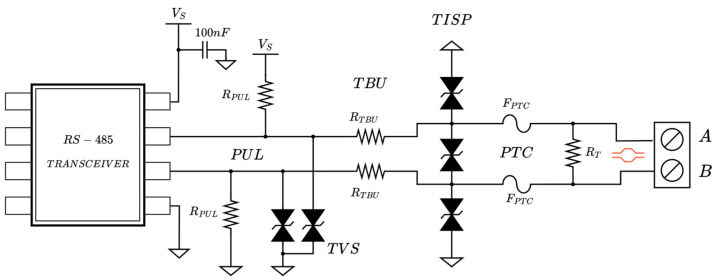
Differential line protection scheme for RS-485 communication.

The organization and setup of the described protections are shown in Figure 5. This circuit is shared by signal pair-based transceivers in the multi-serial bus interface system presented: RS-485, RS-422, and CAN. To support full-duplex RS-485 communication, the circuit must be duplicated.

### 2.3. RS-422

**Figure 6:**
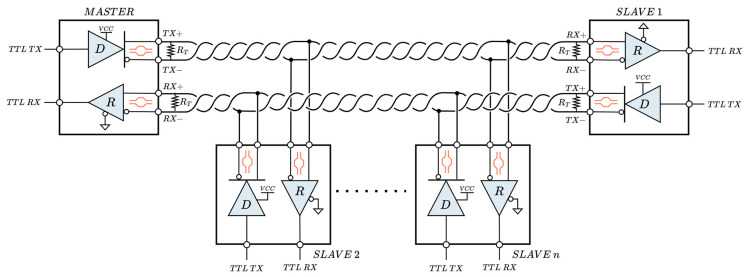
Typical full-duplex RS-422 network with multiple slave devices.

The RS-422 is an interface standard very similar to the RS-485, with the main improvement being the use of two independent balanced differential voltage channels to provide full-duplex communication (TX+/TX− and RX+/RX−). This interface, also known as TIA/EIA-422 and reaffirmed by the Telecommunications Industry Association in 2005 [25], supports various network topologies such as unidirectional, point-to-point, or multi-drop, but no more than one master is allowed in the same network. In contrast to RS-485, the transceiver does not require communication control lines (RE/DE), as both tri-state buffers are always active, with one dedicated to transmission and the other to reception. Figure 6 shows the typical arrangement of an RS-422 network with multiple slaves. It is important to note that each of the two channels, transmit and receive, must be properly terminated at both ends with a termination resistor ($R_{T}$), and that surge protection should be applied to both, as recommended in RS-485.

To implement an RS-422 serial communication bus, two RS-485 transceivers with fixed RE and DE lines are typically used, one dedicated to transmission (TX+/TX−) and the other to reception (RX+/RX−). In this work, such a configuration simplifies the design of the proposed multi-serial bus interface system by allowing a single transceiver to be shared between RS-485 and RS-422 communication modes. Additionally, this setup provides the ability to disable all the tri-state buffers by the available DE/RE control lines, which is essential in the proposed system where the differential lines are shared with other transceivers. RS-422 is the serial interface that requires the highest number of data lines (four) and, therefore, defines the minimum number of pins required for the proposed plug-and-play multi-serial connector.

The electrical signal behavior at the output of the RS-422 differential lines is the same as analyzed in Figure 3 for RS-485, but applied independently for each TX and RX channel. Both channels exhibit similar voltage variations depending on whether they are physically connected or not.

### 2.4. CAN

In contrast to the other selected serial interfaces, CAN not only defines the standard interface but also establishes its own serial communication protocol. The CAN protocol enables efficient and reliable communication in embedded systems, particularly in automotive, industrial automation, and robotics applications.

The physical layer of the CAN interface is similar to that of RS-485, using a twisted-pair cable for carrying the differential signals, labeled CANH (CAN High) and CANL (CAN Low). Logic levels are determined by the voltage difference between these lines: a dominant bit (logic 0) is transmitted when the differential voltage (CANH-CANL) exceeds a positive threshold, while a recessive bit (logic 1) is represented when this voltage difference is close to zero, even negative. As with the RS-485, a 120 Ω termination resistor is required at each end of the bus to prevent signal reflections. However, unlike RS-485, the CAN interface does not rely on shared ground or negative reference between devices, simplifying wiring and improving noise immunity.

The ISO 11898-2 standard [26] defines the physical layer for various versions of the CAN bus, with particular emphasis on the CAN 2.0A protocol, commonly referred to as high-speed CAN. This version supports data transfer rates of up to 1 Mbps. According to the standard, any nominal differential voltage below 0.5 V between CANH and CANL is interpreted as a recessive state, while a differential voltage above 1.5 V is interpreted as dominant. The dominant voltage must be between 1.5 and 3 V, usually around 2 V. Furthermore, the standard does not specify a fixed ground reference; instead, it defines the common voltage of the two signals to float anywhere within the range of −2 V to 7 V.

**Figure 7:**
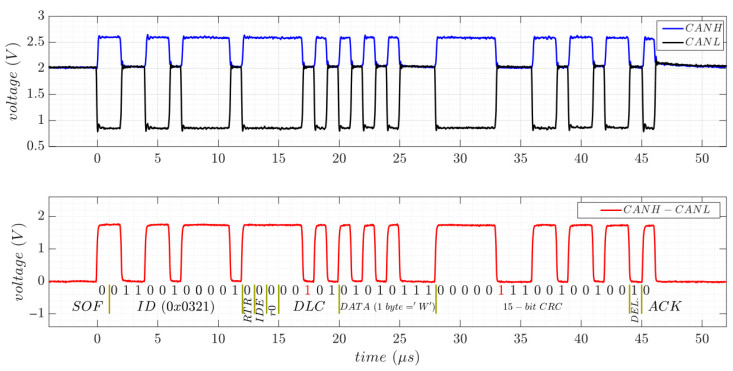
CAN transmission of 1-byte data packet containing the ASCII character “W” using the SN65HVD230 transceiver at 1 Mbps (**top**). Bit-level analysis of the CAN2.0A frame (**bottom**).

Figure 7(top) shows the CANH and CANL signals with respect to ground, along with their differential signal, during the transmission of a 1-byte data packet containing the ASCII character “W” at 1 Mbps. In this test, a 3.3 V SN65HVD230 CAN transceiver from Texas Instruments (Dallas, TX, USA) was connected to an experimental STM32F4-Discovery development board [27], configured for CAN 2.0A at the maximum data rate of 1 Mbps. A 120 Ω termination resistor ($R_{T}$) was used in the setup. In the idle state, both signal lines remain at 2 V, and this voltage stays unchanged during the transmission of recessive bits (CANH-CANL ≈ 0 V). During dominant bits, however, CANH and CANL signals reach 2.7 V and 0.9 V, respectively, resulting in a differential voltage of approximately 1.8 V. Figure 7(bottom) presents a bit-level view of the CAN 2.0A frame, including an 11-bit identifier (ID), a 4-bit data length code (DLC), and a 15-bit cyclic redundancy check (CRC). The stuff bits are highlighted in red.

**Figure 8:**
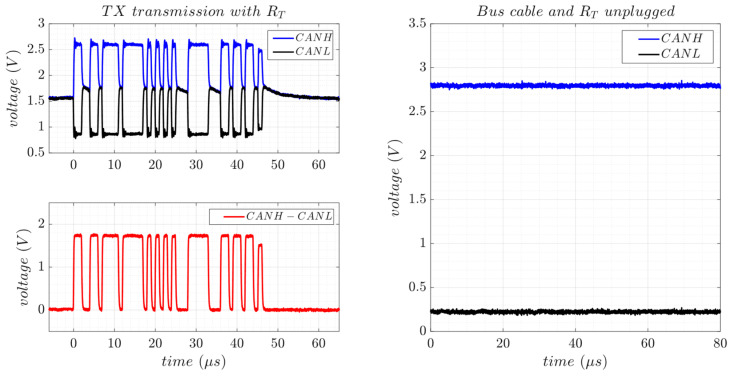
Differential signal output from the CAN transceiver sharing lines with the RS-485 transceiver, including PUL, TISP, and PTC protections. (**Left**) Transmission of the same data packet as in Figure 7. (**Right**) When the bus cable with the $R_{T}$ is unplugged.

To reduce the number of plug-and-play connector pins and simplify the monitoring electronics of the multi-serial system design, CAN and RS-485 differential transmission lines are intended to be interconnected. Figure 8(left) shows the same CAN signal transmission detailed in Figure 7, but with the differential lines now connected in parallel to those of the RS-485 SP3485EN-L transceiver (CANH with A and CANL with B). Besides the termination resistor ($R_{T}$), this setup includes the typical RS-485 protections (PUL, TISP, and PTC), which are shared by both transceivers. The DE/RE control lines of the RS-485 transceiver are configured to disable its tri-state buffers, ensuring that the bus remains unaffected during CAN transmission. By sharing the signal lines and protections with the RS-485, the CAN bus operates unchanged at 1 Mbps. The voltages on the CANH and CANL lines stay the same as in the previous test without shared connections (Figure 7). Unlike the RS-485 transceiver, the CAN transceiver does not include a dedicated line to control the state of receive and transmit buffers; both are always active. As a result, the CAN interface generates an echo on the receive buffer when transmitting, which limits the ability to monitor the bus. Therefore, additional circuitry is required to disable the interface when its differential lines are interconnected with other serial interfaces.

Figure 8(right) shows the CAN differential signals when the bus cable and the $R_{T}$ are unplugged. In this state, the CANH signal rises to approximately 3 V due to the pull-up resistor, while the CANL line drops significantly, reaching almost 0 V. Although these voltage variations clearly distinguish between the connected and disconnected states, they are very similar to those observed for RS-485 (Figure 3(right)). Therefore, additional sensing information from the bus lines is required to differentiate between RS-485 and CAN when they share the same differential pair.

Table 1 presents the average current consumption measured on the differential lines for the RS-485 and CAN interfaces using a standard digital multimeter (AM-555-EUR from BEHA-AMPROBE GmbH, Glottertal, Germany). Measurements were performed under three conditions: (i) with the cable and $R_{T}$ unplugged, (ii) connected to the bus with $R_{T}$ (point-to-point with another transceiver), and (iii) during raw data transmission. The measured current increased substantially when the bus cable with $R_{T}$ was connected; however, the current values remained almost identical for both RS-485 and CAN, consistent with the voltage behavior. Consequently, independent differential signaling is required for these interfaces to automatically determine the connection type when using current and voltage line sensing.

## 3. Hardware Architecture and Development

**Figure 9:**
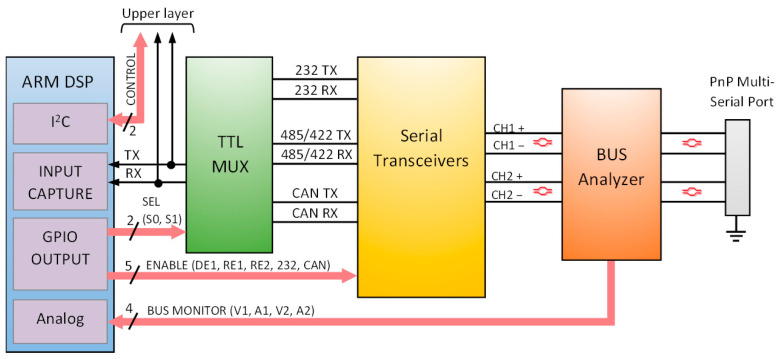
Diagram block of the multi-serial adaptive and monitoring bus system proposed with the three functional stages: TTL multiplexer, Serial Transceivers, and Bus Analyzer.

The hardware integration designed to unify multiple serial interfaces into a single, plug-and-play universal connection with auto-detection, auto-direction control, and monitoring capabilities is organized into three stages, as shown in Figure 9. The first stage (green box) consists of a TTL multiplexer that redirects the TX and RX TTL communication lines from the upper layer (rack concentrator) to the appropriate serial transceiver, depending on the connected interface. The second stage (yellow box), referred to as Serial Transceivers, includes the physical layer transceivers and their interconnection, optimized for a compact external plug-and-play connection. The third stage (orange box) is responsible for protecting the differential signals. It also comprises several analog line sensors for monitoring the physical signals and distinguishing between the different serial interface types. Finally, a dedicated Digital Signal Processor (DSP) (blue box) manages all control lines to select the appropriate interface depending on the analog reading of the line sensors. Additionally, this processor monitors bus activity to detect and set the baud rate, identify bitrate anomalies, and control the direction lines in RS-485 mode. The following subsections describe these hardware stages (Section 3.1, Section 3.2 and Section 3.3), the used DSP (Section 3.4), and the implemented device (Section 3.5).

### 3.1. Serial Transceivers and Bus Unification

**Figure 10:**
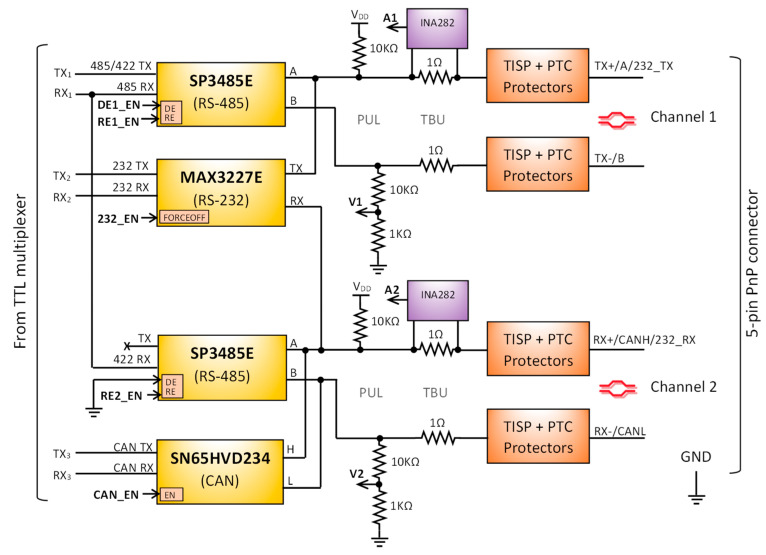
Schematic of the interconnection between the serial interfaces and the shared bus output, including protection elements and the current and voltage line sensors.

Figure 10 shows the transceivers used and their unified interconnection over two shared differential output signal channels. The unification aims to maintain a simplified output pinout, which is one of the key requirements of the proposed device. A minimum of five lines is required for communication, as RS-422 uses two differential signals (four lines), while RS-232 requires an additional dedicated reference ground. These five lines define the simplified 5-pin plug-and-play universal connector proposed.

To support the RS-485 and RS-422, two MaxLinear SP3485E [23] 3.3 V half-duplex RS-485 transceivers have been used. In RS-485 mode, only the transceiver connected to channel 1 is active, and DE1_EN/RE1_EN lines allow for managing the half-duplex direction. For RS-422 mode, both output differential signal channels are necessary: the transceiver on channel 1 is fixed to operate as a transmitter (DE1_EN/RE1_EN: high), while the transceiver on channel 2 is configured as a receiver (RE2_EN: low). From the TTL TX/RX perspective, both interfaces share the same TTL bus, since the TX signal is the same (TX_1_), and the RX signal (RX_1_) only receives data from one channel at a time.

The CAN transceiver used is the SN65HVD234 [28] from Texas Instruments. It complies with ISO 11898-2 standard [26], supports data rates up to 1 Mbps, and integrates fault and ESD protections. This transceiver version includes an additional control pin (CAN_EN) that allows disabling both the driver and receiver tri-state buffers. This feature is essential for maintaining compatibility with the shared output and avoiding interference with other interfaces. The CAN transceiver is placed in the second differential signal channel. This arrangement allows distinguishing between the differential half-duplex connection of RS-485 (channel 1) and the half-duplex connection of CAN (channel 2), since the opposite channel remains disconnected in each case.

The RS-232 transceiver used is the MAX3227E from Texas Instruments [21]. It operates at data rates of 1 Mbps at 3.3 V and supports the TIA/EIA-232-F standard [20]. This transceiver also includes an additional control pin, called FORCEOFF, which disables the internal line driver and receiver from the bus. In RS-232 communication, signals are referenced to ground. This means that both TX and RX lines can only be shared with the positive lines of the differential pairs, where the pull-up resistors are placed. Therefore, the RS-232 driver output (TX) and receiver input (RX) are connected to the positive lines of channels 1 and 2, respectively. In terms of auto-detection, distinguishing RS-232 from other interfaces requires independent line sensors on each line of both differential channels.

### 3.2. Bus Analyzer

The Bus Analyzer (Figure 9 orange box) is the final stage before the output bus connection. It is responsible for protecting the bus and sensing electrical variations in all communication lines. PUL, TBU, TISP, and PTC protections are implemented for both channels 1 and 2 (Figure 10). The Vishay SMAJ12CA bidirectional diode and the Bourns MF-MSMF010-2 resettable fuse, with a trip current of 300 mA, have been selected as TISP and PTC protectors, respectively. For the PUL and TBU protectors, standard resistors are applied and dimensioned to also serve as part of the voltage and current line sensing circuit (Figure 10 PUL and TBU).

The line sensors have been configured under the following key considerations:

1. The sensing component must not interfere with the normal operation of the bus.
2. All communication lines must provide a sensing magnitude.
3. Both current and voltage must be monitored on each differential signal.
4. Avoid redundant information, considering the behavior of the differential signals.
5. The response should be analog within the range supported by the DSP.

To monitor the bus connection and determine the active interface, four current and voltage sensors have been integrated, one voltage and one current sensor per differential communication channel. The negative line of each differential pair includes a voltage sensor implemented using the resistor of the PUL protector as part of a voltage divider composed of 10 KΩ and 1 KΩ resistors (Figure 10). This configuration enables monitoring of voltages up to 36.3 V when using an analog-to-digital converter (ADC) with a 3.3 V reference.

Two additional current sensors are implemented on the positive lines of the differential channels. For this purpose, the Texas Instrument INA282-Q1 [29] current shunt monitor has been selected, offering bidirectional sensing capability with a gain of 50 V/V. These sensors complement the voltage measurement on the negative lines (Figure 10). The design leverages the existing TBU protection resistors as shunt elements. In the current configuration, a 1 Ω shunt resistance is used, allowing a measurement range of $I_{MAX}=\pm 33\;mA$ (Equation (1)), which is adequate for the current levels observed in the experiments. If higher TBU resistance is required, an additional resistor can be added in series with the shunt.

(1) $$I_{MAX}=\pm \;\frac{\frac{VDD}{R_{SHUNT}\cdot GAIN}}{2}=\pm \frac{\frac{3.3}{1\cdot 50}}{2}=\pm \;33\;mA$$

By connecting the external pins REF2 and REF1 of the INA282-Q1 current-sense amplifier to VDD and GND, respectively, the zero-crossing point of the current measurement is shifted to VDD/2, thereby enabling bidirectional sensing. Considering the 12-bit resolution of the ADC used, the current can be calculated using the following formula:

(2) $$I_{CH}=\frac{\left(ADC\_VALUE-2^{\left(12-1\right)}-1\right)\;\frac{VREF}{2^{12}-1}}{R_{SHUNT}\;\cdot \;GAIN}\;=\frac{\left(ADC\_VALUE-2048\right)\;\frac{3.3}{4095}}{1\;\cdot \;50}\;\;[A]$$

Accordingly, the step resolution of the ADC is 16.11 µA, which is suitable for detecting changes in currents of hundreds of microamperes when the interfaces are not transmitting or receiving.

### 3.3. TTL Redirection

In accordance with the objective of unifying serial buses into a single peripheral interface, from the perspective of communication with the upper layer, a redirection of the I/O TTL shared lines is required (Figure 9 green box). The hardware proposed is the 2-channel analog multiplexer/demultiplexer SN74CBTLV3253 [30] from Texas Instruments. This FET-based device provides a 4:1 configuration for each channel, enabling TX to be configured as a multiplexer on one channel and RX as a demultiplexer on the other. Table 2 presents the configuration of the multiplexer control lines, S0 and S1, and the associated configuration of the transceiver enabling lines depending on the serial bus mode to be operated (Figure 10 transceivers). Channel 1A is designated for TTL TX input, while channel 2A is allocated for TTL RX output. It is worth noting that a logical multiplexed selection free (1B4, 2B4) is employed as TTL disconnection, thereby superseding the two additional enabling pins ($1\;\bar{OE}$ and $2\;\bar{OE}$) of the multiplexer. To select an operating mode, a fixed combination of the multiplexer selection lines and transceiver enabling lines must be set. In the case of the RS-485 mode, the control direction lines (DE1_EN, RE1_EN) must be continuously updated according to the current transmission direction.

### 3.4. Digital Signal Processor Hardware Peripherals

**Figure 11:**
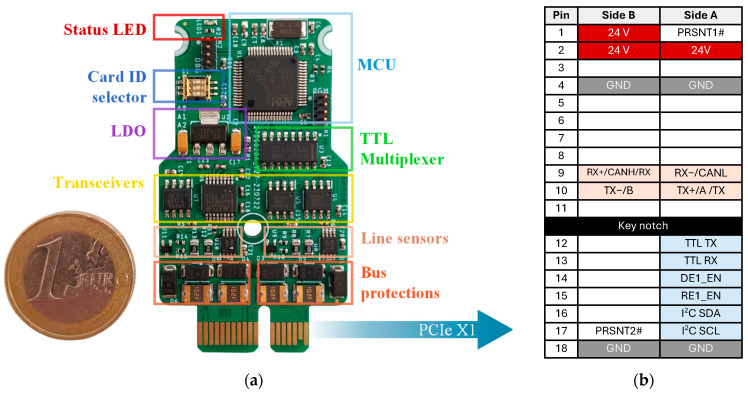
Proposed multi-serial adaptive bus interface card implemented (**a**), and its all-in-one PCIe ×1 connector with the corresponding pinout (**b**). Beige pins correspond to multi-serial port, and blue pins to the upper layer.

The Digital Signal Processor (DSP) integrated into the proposed system is the high-performance 32-bit ARM Cortex-M4 STM32L431RTC6 microcontroller (MCU) from STMicroelectronics [31] (Figure 11 MCU). This microcontroller is well-suited for use as a dedicated DSP, offering an excellent balance of cost, power efficiency, and processing performance. It also includes the necessary hardware peripherals for this proposal, such as an I^2^C interface with slave mode, timers with Input Capture (IC) channels, as well as analog inputs and digital outputs. Figure 9 blue box shows the placement of the microcontroller within the proposed multi-serial system and its relationship with the stages involved.

Two Input Capture (IC) channels of a timer are used to monitor both TX and RX TTL signals by measuring the time intervals between signal edges. This enables the detection of the current pulse width (i.e., baud rate), which is essential for evaluating communication stability. The IC channels are driven by a 16-bit timer running at the CPU clock of 80 MHz, providing a time resolution of 12.5 µs. Additionally, one of the IC channels is used to detect the beginning of an RS-485 half-duplex transmission (falling edge in TX) via a dedicated hardware interrupt, enabling fast switching of the direction control lines.

The main processing algorithm executed by the microcontroller also manages several General-Purpose Input/Output (GPIO) pins, configured as digital outputs, to set the multiplexer selection lines and to enable and disable the communication transceivers. Furthermore, four channels of the internal 12-bit ADC are dedicated to reading analog signals from the voltage and current line sensors of the Bus Analyzer. These analog values are converted into 12-bit digital values through a simple on-demand software conversion that takes only 2.5 cycles at 80 MHz.

Finally, a microcontroller’s I^2^C peripheral interface is configured in slave mode to communicate with the upper-layer processing unit. This upper layer must be responsible for managing all the system functionalities via a register-based protocol. It can manually select an interface or enable the automatic interface detection, activate the RS-485 auto-direction control, enable the baud rate monitor feature, and request communication status and bus fault information.

### 3.5. Card-Based Concept

To develop a smart, modular, and adaptable industrial system with as many serial interfaces as needed, the proposed multi-serial adaptive bus has been implemented using a PCIe card-based architecture, designed to be a part of a rack-mounted module (Figure 1). The concept allows up to eight cards to be connected within a single serial communication (COM) rack, sharing configuration (I^2^C) and communication (TTL TX/RX) lines. This modular architecture guarantees high flexibility to meet the evolving communication requirements of industrial applications, simplifies communication with the upper processing unit, and facilitates maintenance when a multi-bus port fails. Furthermore, simple single-interface serial cards can also be integrated in the same rack alongside the proposed multi-serial bus interface cards, enabling mixed deployment of standard and adaptive ports within a personalized and unified system.

Figure 11a shows the multi-serial adaptive and monitoring bus device implemented as a standalone PCIe-format card with an all-in-one connection. The board measures 60 × 34 mm (including the edge connector) and arranges all the electronic components required for full operation, as described in the previous subsections. It also features the TS2940CW33RPG low-dropout voltage regulator, allowing operation from a 24 V power supply (Figure 11b red pins). In addition, the card includes a user-status LED and a 3-pin DIP switch for setting the card ID, which is used to define the I^2^C device address.

In this modular environment, the serial rack concentrator would provide PCIe sockets to accommodate up to eight multi-serial cards. The rack would expose eight external serial ports, each using a simplified 5-pin connector (e.g., screw terminals, M12, or DB9-style format), where each supported interface maps to a predefined pin assignment, as defined in Table 3. This precise pin mapping is a prerequisite for reliable physical-layer identification.

To handle simultaneous multi-serial port connections, the rack concentrator would require a processor featuring eight TTL-level UART/CAN channels and a shared I^2^C interface. This processor would be responsible for aggregating all communication streams into a unified data bus toward the upper-layer system. For example, a single USB 2.0 link would provide sufficient bandwidth when operating at full load (1 Mbps per 8 channels). In typical deployments, the centralized processing unit is an industrial computer equipped with multiple USB ports, making it possible to operate several serial communication racks in parallel, each populated with multi-serial adaptive interface cards.

## 4. Software Design and Implementation

The multi-serial interface is controlled by a firmware architecture organized into three main functional modules: (1) automatic bus detection and redirection, (2) baud-rate detection and supervision, and (3) RS-485 auto-direction control.

The overall execution is based on independent non-blocking state machines, with each functional module running in parallel on the same microcontroller. The bus auto-detection mechanism determines which serial interface is physically connected, configures the corresponding transceiver, and supervises disconnection and anomalies. Once the interface is established, the baud-rate detection and supervision module monitors real-time communication, while the RS-485 direction-control logic manages direction handling when needed.

### 4.1. Bus Auto-Detection and Redirection

The automatic detection mechanism relies on the voltage and current measurements acquired by the four analog line sensors placed on the differential lines. Variations in signal magnitude over time, compared with predefined thresholds, allow the system to determine which serial standard is connected. The system may operate in either manual or automatic mode. In Manual mode, the upper layer selects the interface explicitly through the I^2^C management bus. In automatic mode, the firmware analyzes the four sensors and autonomously selects and activates the correct serial transceiver.

#### 4.1.1. Three-State Finite-State Machine

**Figure 12:**
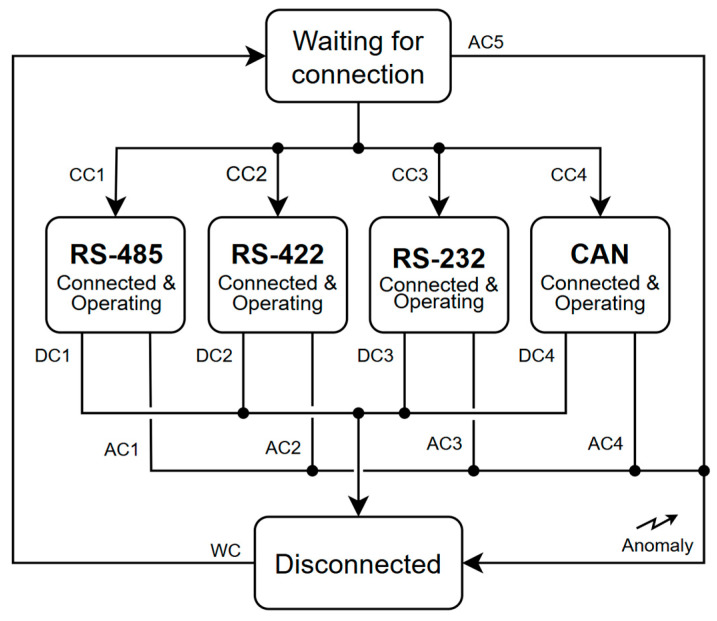
Block diagram of the proposed algorithm for automatic bus detection and redirection.

The automatic detection logic is structured around three main states. Figure 12 summarizes the complete flowchart, the transitions among states, and the associated logical conditions.

- **Waiting for Connection:** All transceivers are disabled while the sensors are monitored for variations indicating the presence of a connected serial device.
- **Connected and Operating:** The transceiver corresponding to the detected serial connection type is active, and the sensors report values within the expected operational range.
- **Disconnected:** The peripheral device has been physically unplugged while the transceiver remains enabled; this state is intended to disable the transceiver and return the system to a state waiting for a new connection.

#### 4.1.2. Logical Sensing Conditions

To transition between states, four logical conditions derived from the sensors are defined. A minimum stabilization time of one second is stabilized for any logical condition to be validated before allowing a state transition.

- **Connection Condition (CC):** Used to detect the presence of a valid serial connection. Four mutually exclusive CC variants (Figure 12, CC1 to CC4) correspond to RS-485, RS-422, RS-232, and CAN, respectively.
- **Disconnection Condition (DC):** Identifies the physical removal of the device while the corresponding interface remains active.
- **Waiting Condition (WC):** Characterizes the absence of any connected peripheral, with all interfaces disabled.
- **Anomaly Condition (AC):** Triggered when any sensor exhibits voltage or current values outside its normal operational range.

The DC and AC depend on the serial interface connected and are specific to each interface mode. If an anomaly persists for one second, the system notifies the upper layer via I^2^C and forces a transition to the DC state to disable the affected transceiver.

#### 4.1.3. Thresholds Extraction from Experimental Measurements

To define the logical conditions required by the state machine, a comprehensive communication test was performed for all supported interfaces. Sensor readings were acquired during the following sequence: (1) plugging in the peripheral device, (2) powering it on, (3) enabling the transceiver, (4) transmitting and receiving raw data and (5) unplugging the device while keeping the transceiver active.

Table 4, Table 5, Table 6 and Table 7 report the current and voltage fluctuations observed during these steps for RS-485, RS-422, RS-232, and CAN, respectively. Table 8 provides the baseline readings when no device is connected and all transceivers are disabled, which define the Waiting Condition (WC).

The communication tests were conducted using a point-to-point configuration. The serial peripheral devices employed included the DSD TECH SH-U10L USB-to-RS-485 adapter [32] and the DSD TECH SH-U11 USB-to-RS-422 adapter [33], both based on MaxLinear SP3485E chipsets; the UGREEN USB-to-RS-232 adapter [34], featuring the Prolific PL2303 chipset; and the SN65HVD230 CAN bus transceiver from Texas Instruments along with a STM32F4-Discovery board configured for raw data transmission. An additional STM32F4-Discovery board, equipped with TTL UART and CAN hardware peripherals, was used as the upper-layer communication unit of the presented architecture.

The transmission test involved sending random raw data bytes for 10 s at the maximum supported speed of 1 Mbps. For differential signaling serial connections, a 120 Ω termination resistor should be included as part of the peripheral device to improve sensor-level differentiation among interface types. The sensor readings were performed via software with an acquisition sampling rate of 100 ms.

#### 4.1.4. Logical Conditions Definition

Considering the line sensor values obtained in Table 8, when the system is in Waiting for Connection state, the Waiting Condition (WC) is defined in Equation (3), where voltage is in volts and current in microamperes:

(3) $$WC=V1<0.50\;\;\&\;V2<0.50\;\&\;\left(-40<A1<40\right)\;\&\;(-40<A2<40)$$

Due to potential variations in the zero-crossing of the analog current sensors, a factory calibration is required, assuming that no serial device is connected. During this calibration, the A1 and A2 sensor values are stored in internal flash memory and later used as offset corrections for subsequent readings.

The sensor values obtained enable the distinction between interfaces. The Connection Conditions (CC) established for each interface are as follows:

(4) $$\begin{array}{c} CC1=V1>0.50\;\;\&\;\;V2<0.50\;\;\&\;\;A1>60\;\;\&\;\;A2<40\\ \;CC2=V1>0.50\;\;\&\;\;V2>0.50\;\;\&\;\;A1>60\;\;\\ \;CC3=V1<0.50\;\;\&\;\;V2<0.50\;\;\&\;\;A1>60\\ \;CC4=V1<0.50\;\;\&\;\;V2>0.50\;\;\&\;(-50<A1<50)\;\;\&\;\;A2>6 \end{array}$$

In the case of CC3, the RS-232 device must be powered to detect its connection, whereas the other interfaces are detected as soon as they are plugged in. Furthermore, certain RS-232 devices may not be detected despite being physically connected and powered because they remain in standby mode until they begin transmitting.

The Disconnection Conditions (DC) defined for each interface are detailed in Equation (5). Each interface has a specific DC considering that the interface could remain on, and the only detectable sensor variations result from the physical device disconnection.

(5) $$\begin{array}{c} DC1=V1<0.50\;\;\&\;\;V2<0.50\;\;\&\;\;A1>60\;\;\&\;\;A2<60\\ \;DC2=V1<0.50\;\;\&\;\;V2<0.60\;\;\&\;\left(-60<A1<60\right)\;\&\;(-60<A2<60)\;\;\\ \;DC3=\left(-60<A1<60\right)\;\&\;(-60<A2<60)\;\;\\ \;DC4=V2<0.70 \end{array}$$

The Anomaly Conditions (AC) established are detailed in Equation (6). The anomaly is identified when a not-used sensor or a used but stable sensor value, during normal operation, presents values outside the expected behavior.

(6) $$\begin{array}{c} AC1=V2>0.60\;\left|\;\left(A1<-100\;\right|\;A1>100\right)\;\left|\;\left(A2<-25,000\;\right|A2>25,000\right)\\ \;AC2=A1<-25,000\;\left|\;\left(A2<-25,000\;\right|A2>25,000\right)\\ \;AC3=V1>0.50\;\left|\;V2>0.60\;\right|\;\left(A1<-25,000\;\right|\;A1>25,000)\;|\;\left(A2<-25,000\;\right|\;A2>25,000)\\ \;AC4=V1>0.50\;\left|\;\left(A1<-200\;\right|\;A1>200\right)\;\left|\;\left(A2<-25,000\;\right|A2>25,000\right)\\ \;AC5=V1>5\;\left|\;V2>5\;|\;\left(A1<-20,000\;\right|\;A1>20,000\right)\;\left|\;\left(A2<-20,000\;\right|A2>20,000\right) \end{array}$$

### 4.2. Baud Rate Detection and Supervision

A baud rate analyzer capability is integrated into the dedicated DSP, with the primary objective of enabling automatic baud rate configuration when an external device is connected and monitoring communication quality in real-time, reporting anomalies that affect data integrity. The methodology used involves extracting the bit rate by analyzing the edges of both TTL TX and RX signals during transmissions between the upper-layer unit and the connected serial peripheral device. The transmitting and receiving bit rates are continuously obtained and updated by measuring the time interval between consecutive signal edges. Two dedicated Input Capture (IC) channels of a microcontroller’s timer are responsible for detecting edge changes as quickly as possible using hardware.

The timer used is a 16-bit timer operating at a frequency of 80 MHz, which provides a counting resolution of 0.0125 µs and reloads every 819.2 µs. Upon detecting an edge on the IC channel, the current counter value is frozen, and an IC interrupt is generated. The interrupt service routine calculates the elapsed time since the previous edge and subsequently determines the current bit rate. The number of timer overflows occurring between two detected edges is counted in the timer update interrupt and used to correct the timing calculation. Therefore, there are no detection limits for lower bit rates. However, at bit rates exceeding 0.5 Mbps (2 µs between edges), the microcontroller begins to experience overlapping IC interrupts. To address this issue and ensure reliable detection at bit rates of at least 1 Mbps, only falling edges are considered. Likewise, this falling-edge detection is shared with the automatic direction control lines feature of the RS-485 interface, described in the following subsection.

**Figure 13:**
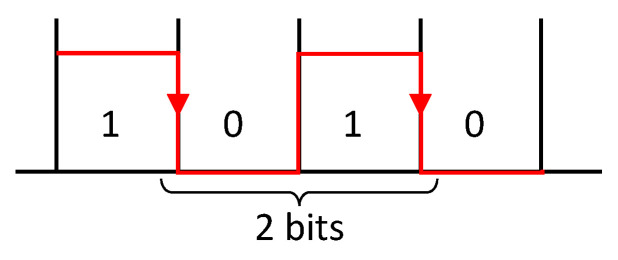
Communication bit sequence to determine $T_{1}$.

The minimum count value ($T_{1}$) between two consecutive falling edges, illustrated in Figure 13, is determined based on the bits per second (BPS) of the communication and the timer frequency (80 MHz) using the following expression:

(7) $$T_{1}\left[counts\right]=\frac{80\left[MHz\right]\cdot 2\;\left[bit\right]}{BPS\;\left[\frac{bits}{s}\right]}=\frac{160\cdot 10^{6}\left[\frac{counts}{s}\cdot bit\right]\;}{BPS\;\left[\frac{bits}{s}\right]}=\frac{160\cdot 10^{6}}{BPS}\left[counts\right]$$

**Figure 14:**
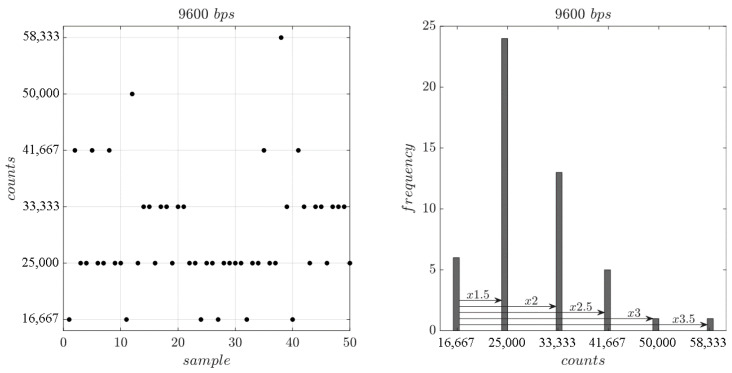
Timer counts computed between consecutive falling edges during a serial ASCII data transmission at 9600 bps. Count values for 50 samples (**left**) and frequency of the count values obtained (**right**).

Figure 14(left) shows the timer counts computed between consecutive falling edges during a serial transmission for random ASCII data at 9600 bps. The resulting counts are always multiples of $\frac{1}{2}T_{1}$, specifically $T_{1}\cdot \left\{1,\;1.5,\;2,\;2.5,\;3,\;3.5\right\}$. Their value depends on the number of consecutive logical zeros followed by ones that are between falling edges. Figure 14(right) shows the frequency of the counter values obtained between edges after 50 samples. The maximum value obtained is $3.5\cdot T_{1}$, corresponding to the maximum possible of 7 bits between falling edges in ASCII encoding. Its occurrence is only 2% of the total counter values obtained. Values exceeding this maximum are negligible and, therefore, not addressed in the proposed detection method.

Table 9 presents the theoretical count values between falling edges for the standard bit rate used in the industry with up to 1 Mbps and a maximum of ${3.5\cdot T}_{1}$ count values. There are bit rates that share the same counter values (in red), for instance, the ${1\cdot T}_{1}$ at 2400 bps is the same as ${2\cdot T}_{1}$ at 4800 bps. This reflects that it is not possible to distinguish between baud rates with only one sample. The ${2.5\cdot T}_{1}$ and ${3.5\cdot T}_{1}$ columns do not present ambiguities, but their occurrence is exceedingly rare (10% and 2%, respectively) and do not guarantee robustness and speed on detection. In terms of baud rate accuracy, the test presented in Figure 14 has been repeated for all baud rates listed in Table 9, and the relative error of the counts computed, compared with the theoretical value presented in Table 9, was always below 0.36% for all multiples of $T_{1}$.

**Figure 15:**
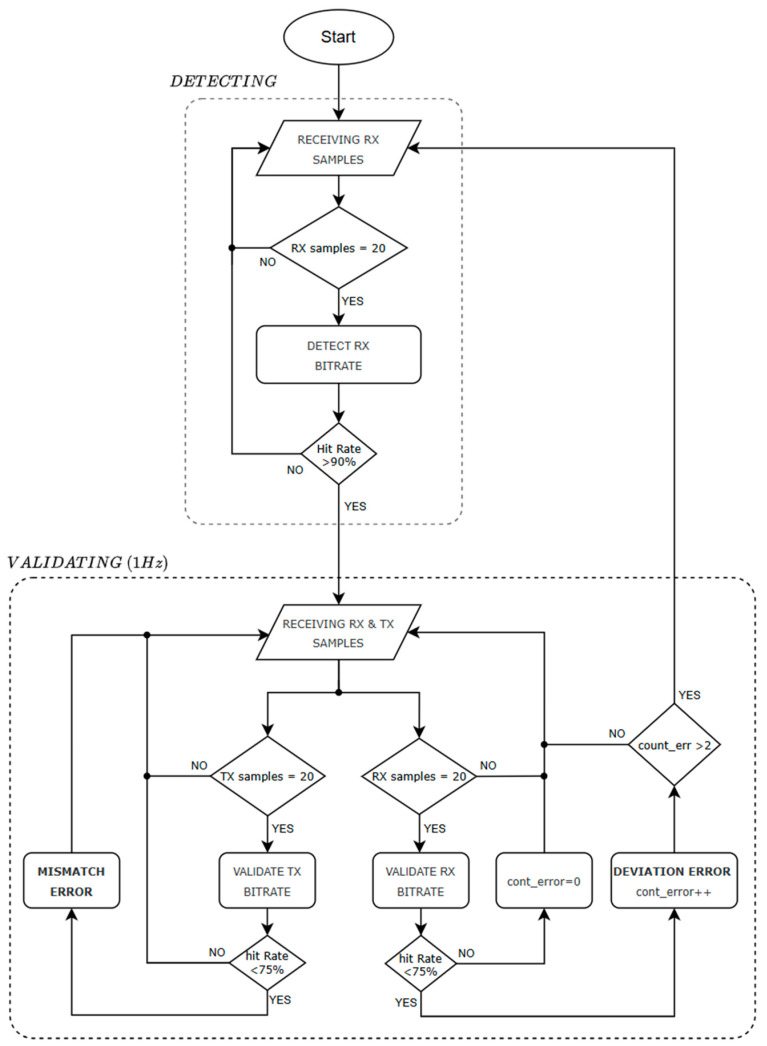
Flowchart of the baud rate detection and validation algorithm presented.

The proposed baud rate detection method is based on checking a set of consecutive timer count values between falling edges (20 samples) with the values defined in Table 9. The baud rate is considered detected when more than 90% of values match with the multiples of $T_{1}$ corresponding to one of the baud rates listed in Table 9, within a deviation margin of ±1%. Figure 15 details the flow diagram of the baud rate detection and supervision procedure. The first stage consists of detecting the current receiving baud rate. When a set of 20 receiving samples matches the values of one baud rate of Table 9, the receiving baud rate is established and begins with the validating stage that supervises problems of baud rate mismatch or deviation. The following key considerations should be noted when using this detection method:

- Supports only the fixed baud rates listed in Table 9. Increasing the number of supported baud rates results in higher memory usage and longer computation time.
- Requires initial communication from the connected serial device before detecting its baud rate.
- Detection accuracy depends on the content of the transmitted data. In atypical scenarios where the transmission consists of fixed or repetitive data patterns with minimal bit transitions, the detection could be delayed, incorrect, or multiple false matches. In such cases, the algorithm will recover when varied values of data are transmitted since it is under constant supervision.
- Data transmissions typically include breaks between bursts of bytes, which produce timer counter values exceeding the expected range (>3.5$\cdot T_{1}$). These values are discarded along with their subsequent valid sample, as the latter is also inaccurate.
- In CAN communication, the transceiver generates an echo on the RX line while transmitting data. Thereby, falling-edge detection on the RX line is disabled, and consequently, mismatch supervision is not applied in this mode.

Once the baud rate of the connected serial device is detected, the validation stage continuously acquires timer count samples between edges for both TX and RX directions (Figure 15). For RX samples, if less than 75% of 20 consecutive samples fall outside the expected values (Table 9) for the current detected baud rate, and this condition occurs more than twice, the DEVIATION anomaly is triggered and reported to the upper layer. The algorithm is then restarted. For RX samples, if the hit rate, defined as the number of samples matching the expected values for the receiver’s baud rate, falls below 75%, the MISMATCH anomaly is triggered and also reported to the upper layer. This error remains active until the upper layer adjusts its baud rate to match that of the connected serial device.

### 4.3. RS-485 Auto-Direction Control Lines

In centralized communication systems, where several RS-485 interfaces are present and managed by a single processor unit, the direction control lines can introduce significant challenges in terms of processing complexity, response time, and hardware interconnection. To address these issues, the proposed device leverages its onboard microcontroller to implement a decentralized automatic control mechanism for the RS-485 direction lines.

The presented device supports both manual and automatic RS-485 direction control, configurable via the dedicated I^2^C bus. In automatic mode, the DE1_EN and RE1_EN lines are exclusively managed by the device with a push-pull configuration. These control lines remain in receiving state (DE1_EN: low, RE1_EN: high) until a start of transmission is detected on the TTL TX signal. The falling edge detector triggers an interrupt service routine (ISR) where the control lines are promptly switched to transmitting state (DE1_EN: high, RE1_EN: high).

**Figure 16:**
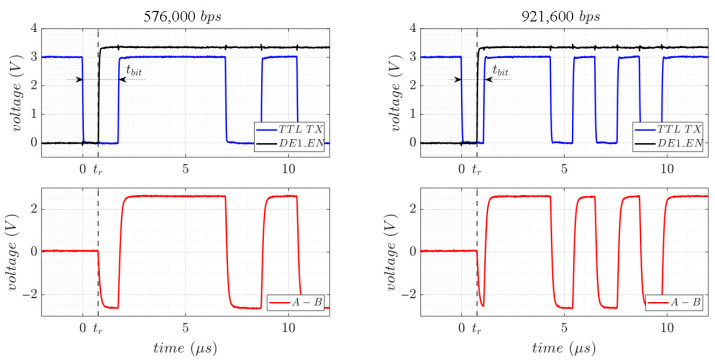
Start of an RS-485 transmission of the ASCII character “W” at 576,000 bps (**left**) and at 921,600 bps (**right**) using the auto-direction control line system.

Figure 16 illustrates the RS-485 TTL and differential signaling at the beginning of the transmission of the ASCII character “W” at 576,000 bps and 921,600 bps using the proposed auto-direction control method. The DE1_EN line is updated with maximum priority at the beginning of the ISR using inline assembly code to ensure minimal reaction time. With the selected 80 MHz microcontroller, the measured reaction time was $t_{r}$ = 751 ns, which is sufficient to detect the first transmitted bit (start bit) of the transmission for baud rates up to 576,000 bps (Figure 16(left)) where $t_{bit}$ = 1736 ns. However, at higher baud rates where the $t_{bit}$ < 2$\cdot t_{r}$, the start bit is missed and, consequently, the payload of the first byte becomes corrupted (Figure 16(right)). To achieve reliable operation data rates up to the supported by the transceiver of 1 Mbps with automatic direction control lines, a faster microcontroller or additional dedicated hardware (such as a precision timer [35]) should be required.

In automatic mode, the receiving state must be recovered after some time of inactivity in the TTL TX signal. When the elapsed time from the last detected falling edge reaches a predefined threshold, $t_{end}$, the control lines are automatically switched to receiving state. The value of t_end is calculated considering the worst-case scenario: continuous transmission of logic zeros with the minimum baud rate of 300 bps including all possible protocol control bits. Under these conditions, $t_{end}$ must be equal or greater than the time of transmitting 12 bits (1 start + 8 data + 1 parity + 2 start) at 300 bps, $t_{end}\geq 40\;ms$. Also, this latency can be optimized via an I^2^C command, which adapts $t_{end}$ to the actual bit duration $t_{bit}$, estimated in real time using the baud rate detection algorithm described in the previous section.

## 5. System Validation

**Figure 17:**
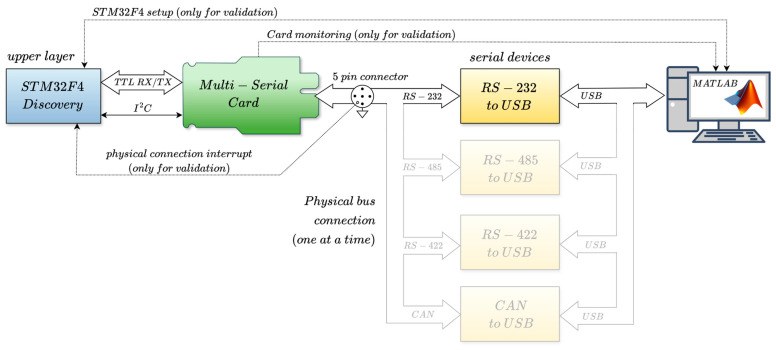
Comprehensive overview of the system setup used in the experimental validation.

The system presented in this work was tested under various communication scenarios to evaluate its performance in terms of interface detection, interface redirection, data transmission, and baud rate detection and supervision. Figure 17 illustrates the experimental setup, including all devices and interconnections used during testing. The upper-layer communication device, responsible for managing the proposed multi-serial unit and generating the serial communication, was an STM32F4-Discovery board. It was configured to operate with the UART and CAN hardware peripherals at speeds up to 1 Mbps. A raw data transmission and reception mode is also implemented internally, along with the control of an external interrupt (EXTI) pin, which enabled detection of physical connections to measure the system’s response time.

The serial devices used to verify connectivity and communication with the upper layer were the same as those described in Section 4.1. The RS-485, RS-422, and RS-232 USB serial adapters were connected to a PC as USB-CDC virtual COM ports and were controlled via MATLAB (R2025b) software to perform specific data transmissions and subsequent analysis. For the CAN network, a PCAN-USB interface from PEAK-System Technik GmbH (Darmstadt, Germany) [36] was employed. This device was also managed from MATLAB, using the Vehicle Network Toolbox, to transmit and receive specific data at speeds up to 1 Mbps.

### 5.1. Auto-Detection and Redirection

The bus auto-detection and redirection capability was validated by repeatedly connecting and disconnecting the proposed device from the serial bus 20 times. Table 10 presents the success rate of detection and redirection for each serial interface, along with the corresponding average detection times. All trials yielded satisfactory results, demonstrating that the proposed system accurately detected and redirected the interface connections.

**Figure 18:**
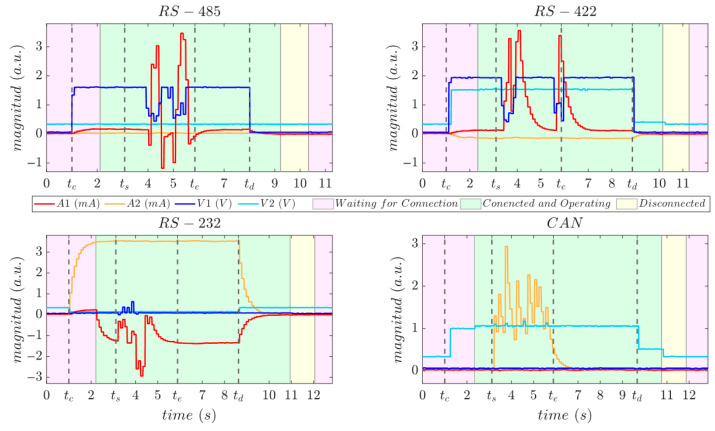
Evolution of analog line sensors values and algorithm state (background color) during connection ($t_{c}$), transmission (from $t_{s}$ to $t_{e}$), and disconnection ($t_{d}$) for each supported interface.

Figure 18 shows the evolution over time of the four analog line sensors together with the algorithm status during the connection and disconnection test for one representative trial of each supported interface. During this real-time acquisition, the user physically connects the device (at $t_{c}$), after which the algorithm detects and redirects the interface and transitions to the Connected and Operating state (Figure 18, green area). Then, the upper-layer (STM32F4-Discovery board) transmits blocks of 256-byte random data for 3 s (from $t_{s}$ to $t_{e}$). Finally, the user disconnects physically the device (at $t_{d}$), causing the algorithm to transition to Disconnected state (Figure 18, yellow area) and subsequently to the Waiting for Connection state (Figure 18, purple area), preparing the interface for a new connection. The background colors in Figure 18 indicates the real-time state of the algorithm during the acquisition.

An average time of approximately 1.2 s was taken across all interfaces to complete the connection procedure and make the medium ready for data transmission. This delay is dominated by the intentioned 1-s stabilization window, required for a logical condition to be considered valid before transition to a new state. In the connection procedure, at least 1 s was taken for validating the CC condition to trigger the transition from the Waiting for Connection state to the Connected and Operating state. Although the sensing hardware (voltage and current sensors) responds significantly faster in all interfaces (see Figure 18, after the physical connection $t_{c}$), the stabilization margin was deliberately oversized to increase robustness and avoid false transitions in real-world deployments.

The disconnection procedure requires passing through DC and WC conditions before being ready to accept a new connection. Results show that the time required is at least 2 s (one per state). The worst result was for RS-232 communication, where the disconnection time increased to 3 s. This is because of the RS-232 disconnect condition (DC3) requires very low A1 and A2 sensor values (below 60 µA), and they exhibit slow falling response (Figure 18, RS-232 after $t_{D}$) due to the transceiver behavior.

The disconnection delay must be observed before connecting a new serial device; otherwise, the system may remain locked in the DC condition and fail to detect the subsequent connection. In real applications, the physical connection or disconnection of a serial device typically occurs only during the initial setup or maintenance phase, not during real-time operation. Therefore, the system is not intended to switch communication standards dynamically at runtime, but rather to detect the correct interface once at system initialization (or when a device is physically replaced).

### 5.2. Transmission Data Integrity

A data transmission integrity test was carried out between the upper-layer unit (STM32F4-Discovery) and each of the peripheral devices used to validate the four serial interfaces. A predefined 10 Kbyte random data sequence was transmitted in both sending and receiving modes across each interface. The data sequence was known at both ends and, upon reception, was compared bit by bit with the expected values. The test was repeated at several standard baud rates (listed in Table 9), up to 1 Mbps. All transmissions succeeded in both directions, with no bit-level errors detected.

### 5.3. Baud Rate Detection and Monitoring

**Figure 19:**
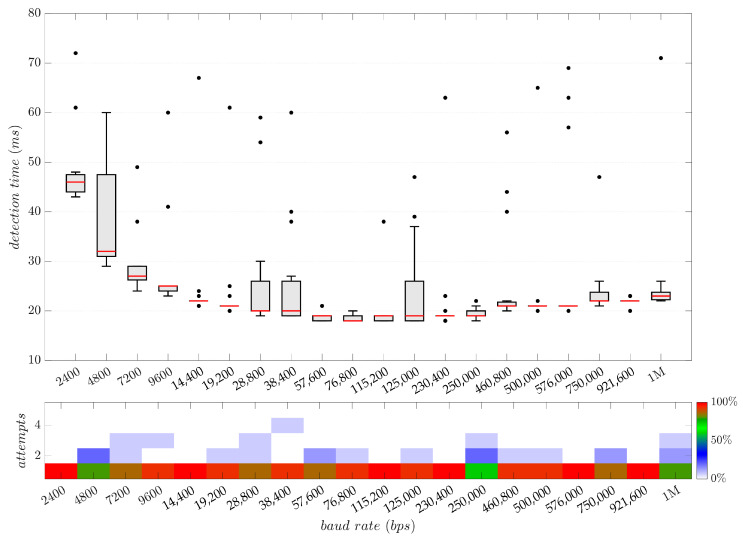
Box plot of the baud rate detection time using the algorithm presented in Figure 15 across 15 trials for each of the supported speeds during a raw random data transmission (**top**). Distribution of 20-sample block validation attempts required for successful detection (**bottom**).

The baud rate detection capability was tested across different baud rates configurations by receiving random raw data from a RS-232 serial peripheral connected. The external serial device continuously transmitted random data to the upper-layer communication unit, while the algorithm proposed in Figure 15 analyzed the TTL RX signal to determine the baud rate in use. This procedure was repeated 15 times for each of the 20 standard supported baud rates (Table 9). In all trials, the detection result was successful, and no false detections occurred. In 91.6% of the trials, the baud rate was correctly identified within the first set of 20 rising-edge samples acquired (one algorithm attempt, Figure 19(bottom)). These results are attributed to the presence of continuous signal transitions (rising and falling edges) in the random data stream. Considering the successful results obtained in the data transmission integrity tests and given that the analysis is performed at the TTL level, the baud rate detection performance is expected to be identical for all other supported serial transceivers. The same applies to the second algorithm stage, where the system monitors the current baud rate using both TTL RX and TX data streams to detect deviation and mismatch errors, as it employs the same detection procedure as in the first detection stage.

Figure 19 illustrates the box plot of the baud rate detection time during random data transmission across 15 trials for each of the supported speed configurations. The baud rate detection algorithm employed utilizes IC interrupts to measure the elapsed time between edges; however, the core algorithm executes within the primary CPU loop, influencing its detection response time. It is important to note that CPU resources are also allocated to the line sensors monitoring task and the automatic bus interface detection and redirection state machine. Despite these factors, the results indicate that the response time of the baud rate algorithm remained below 80 ms in all cases.

For baud rates below 19,200 bps, the detection time varies according to transmission speed, increasing as the baud rate decreases. For baud rates between 28,800 and 250,000 bps, the detection time reaches its minimum value of 18 ms, constrained by the available MCU resources during the algorithm updates in the main loop. At baud rates greater than 250,000 bps, the detection time tends to rise again, owing to the hardware performance limitations that generate IC interrupts overruns. The loss of some pending IC interrupts is not deemed critical for the baud rate detection, as successful detection was maintained at baud rates up to 1 Mbps. This is attributable to the fact that the IC channel timer remains continuously active, and subsequent handled interrupt also computes a valid count value multiple of $T_{1}$, provided it does not exceed the maximum verification value established of 3.5 times $T_{1}$.

## 6. Conclusions

This work presents a unified hardware architecture capable of merging RS-232, RS-485, RS-422, and CAN into a simplified 5-pin plug-and-play interface, enabling interoperability across heterogeneous serial technologies without relying on mechanical relays. By implementing the switching at the TTL level through a single logical multiplexer, the proposed solution minimizes hardware complexity and requires only one shared TTL interface on the upper-layer processor.

A key outcome of this research is the development of a non-intrusive sensing strategy that repurposes the bus protection components to monitor current and voltage variations on the communication lines. This approach preserves the electrical integrity of all supported interfaces while providing reliable data for automatic identification of the connected bus type. Combined with the defined logical rules, this sensing mechanism enabled consistent interface detection and correct bus redirection in all evaluated scenarios.

This work also integrates an automatic direction-control mechanism for half-duplex RS-485 communication, running on the same microcontroller and eliminating the need for external timing circuits or manual control. Additionally, a real-time baud-rate detection and supervision algorithm was implemented using the processor’s internal timer resources. This method accurately determined standard baud rates up to 1 Mbps and provided continuous detection of mismatches and deviations, improving communication reliability in dynamic environments.

All these functions (bus sensing, protocol identification, line redirection, baud-rate detection and supervision, and RS-485 direction control) were consolidated into a low-cost (≈10 €) PCIe card. The resulting module forms part of a scalable rack-mounted communication system that supports seamless integration of serial devices from different manufacturers while reducing cabling requirements and eliminating the need for manual configuration. By automating interface selection and communication-parameter setup, the proposed solution accelerates system deployment, decreases the likelihood of operator errors, and contributes to more flexible and adaptive industrial communication infrastructures.

## 7. Limitations and Future Work

The proposed system currently supports auto-detection only for the 20 most common industrial baud rates. Unfortunately, it does not operate correctly with uncommon or proprietary baud rates. This limitation could be addressed by adding a new feature to the system, allowing the upper layer to insert non-standard baud rates into the system’s baud-rate table (Table 9) through I^2^C.

Similarly, the system cannot determine the baud rate of an external device that does not transmit a minimum amount of data. For such devices, the upper layer can set the baud rate and disable both the autodetection and supervision mechanisms. This special case was considered in the present study, and the solution has already been implemented.

Another potential limitation arises from the use of fixed voltage and current thresholds to define the Connection, Disconnection, Waiting, and Anomaly conditions. All experiments in this work were conducted in a controlled environment, using cable lengths between 20 and 100 cm. In real deployments, factors such as temperature variations, longer cables, electromagnetic interference, and component aging may push sensor readings outside the predefined ranges. Addressing this issue would require firmware enhancements to support an auto-calibration mechanism. Possible approaches include: a) allowing the upper layer to issue an I^2^C command to trigger a calibration routine that updates the thresholds for specific transceiver based on live sensor readings; or b) automatically recalibrating these thresholds periodically (e.g., once per hour) while the system is operating normally and without transmitting.

The RS-485 auto-direction control lines function reliably up to 570,000 bps due to MCU hardware limitations. To reach 1 Mbps, we propose two alternatives: (a) using manual mode and delegating direction-line control to the upper layer; or (b) employing a faster (though more expensive) MCU capable of handling data rates of 1 Mbps or higher.

Finally, although the proposed device aims to offer plug-and-play operation, the external device’s wiring must be connected to the rack’s 5-pin concentrator in a specific configuration (see Table 3). Standard pinout configurations, such as those used with DB9, cannot support a multi-serial interface due to electrical incompatibilities between lines.

## Figures and Tables

**Table 1:** Average current consumption measured on the differential signals of the CAN and RS-485 transceivers, including PUL, TISP, and PTC protections.

Transceiver	Signal	Unplugged (µA)	Plugged in with the $R_{T}$ (µA)	Transmitting Data (µA)
SN65HVD230	CANH	0	294	295
	CANL	0	291	321
SP3485E	A	0	321	338
	B	0	335	336

**Table 2:** Configuration of the SN74CBTLV3253 multiplexer and transceiver enabling lines for activating a specific serial interface mode.

	TTL Redirection				Transceiver Enabling Lines				
Mode	S1	S0	TX (1A)	RX (2A)	DE1_EN	RE1_EN	RE2_EN	232_EN	CAN_EN
RS-485	L	L	1B1: TX_1_	2B1: RX_1_	Half-duplex dir.		H	L	L
RS-422	L	L	1B1: TX_1_	2B1: RX_1_	H	H	L	L	L
RS-232	L	H	1B2: TX_2_	2B2: RX_2_	L	H	H	H	L
CAN	H	L	1B3: TX_3_	2B3: RX_3_	L	H	H	L	L
Disabled	H	H	1B4: Free	2B4: Free	L	H	H	L	H

**Table 3:** 5-pin connector mapping depending on the external interface type connected.

Rack Connector Pin	PCIe Card Pin	R-485	RS-422	RS-232	CAN
1	10A	A	TX+	TX	-
2	10B	B	TX−	-	-
3	9B	-	RX+	RX	CAN H
4	9A	-	RX−	-	CAN L
5	18A, 18B, 4A, 4B	-	-	GND	-

**Table 4:** Line sensor variations (minimum and maximum) acquired during a complete communication procedure with an RS-485 peripheral device.

Step	Description	MUX Mode	A1 Sensor (µA)	V1 Sensor (V)	A2 Sensor (µA)	V2 Sensor (V)
1	Device plugged in	Disabled	+173/180	1.59/1.60	−1/+4	0.33/0.34
2	Plugged in and powered	Disabled	+160/170	unchanged	unchanged	unchanged
3	Transceiver activated	RS-485	+176/190	1.64/1.65	unchanged	unchanged
4	Transmitting to the device	RS-485	−20,400/−21,760	unchanged	unchanged	unchanged
5	Receiving from the device	RS-485	−1700/−4020	1.05/1.80	unchanged	unchanged
6	Unplugged	RS-485	+15/22	0.07/0.08	unchanged	unchanged

**Table 5:** Line sensor variations (minimum and maximum) acquired during a complete communication procedure with an RS-422 peripheral device.

Step	Description	MUX Mode	A1 Sensor (µA)	V1 Sensor (V)	A2 Sensor (µA)	V2 Sensor (V)
1	Device plugged in	Disabled	+206/214	1.27/1.28	−13/+23	1.03/1.04
2	Plugged in and powered	Disabled	+126/134	1.91/1.92	−165/−175	1.52/1.53
3	Transceiver activated	RS-422	+20,532/20,553 ^1^ +130/147 ^2^	0.12/0.13 ^1^ 1.91/1.92 ^2^	−192/−208	1.53/1.54
4	Transmitting to the device	RS-422	−4456/−9825	0.55/1.17	−165/−175	1.51/1.55
5	Receiving from the device	RS-422	+126/134 ^1^	1.91/1.92 ^1^	−4754/−6473	1.51/2.48
6	Unplugged	RS-422	+19/27	0.07/0.08	−5/+5	0.41

^1^ TX always on. ^2^ TX active only when transmitting.

**Table 6:** Line sensor variations (minimum and maximum) acquired during a complete communication procedure with an RS-232 peripheral device.

Step	Description	MUX Mode	A1 Sensor (µA)	V1 Sensor (V)	A2 Sensor (µA)	V2 Sensor (V)
1	Device plugged in	Disabled	+9/13	0.00/0.01	−7/+5	0.33/0.34
2	Plugged in and powered	Disabled	+226/235	unchanged	−3475/3508 ^1^	unchanged
3	Transceiver activated	RS-232	−1423/−1438	0.10/0.11	unchanged	0.14/0.15
4	Transmitting to the device	RS-232	−15,234/−19,024	0.06/0.11	3445/3491	unchanged
5	Receiving from the device	RS-232	−1380/−1450	0.10/0.11	−7230/−11,344	unchanged
6	Unplugged	RS-232	−11/+5	0.00/0.02	−9/+5	0.33/0.34

^1^ 0 µA after some seconds (standby).

**Table 7:** Line sensor variations (minimum and maximum) acquired during a complete communication procedure with a CAN peripheral device.

Step	Description	MUX Mode	A1 Sensor (µA)	V1 Sensor (V)	A2 Sensor (µA)	V2 Sensor (V)
1	Device plugged in	Disabled	+7/16	0.00/0.01	+10/20	0.88/0.89
2	Plugged in and powered	Disabled	unchanged	unchanged	+13/22	unchanged
3	Transceiver activated	CAN	unchanged	unchanged	+8/16	1.07/1.08
4	Transmitting to the device	CAN	+14/34	0.07/0.08	+5840/9640	1.16/1.23
5	Receiving from the device	CAN	+48/57	0.06/0.07	−258/+243	1.19/1.32
6	Unplugged	CAN	unchanged	unchanged	−7/+4	0.52/0.53

**Table 8:** Minimum and maximum line sensor values acquired during the Waiting Connection state, with no device plugged in.

A1 Sensor (µA)	V1 Sensor (V)	A2 Sensor (µA)	V2 Sensor (V)
−5/+16 **µ**A	0.00/0.02	−1/+9 **µ**A	0.33/0.34

**Table 9:** Theoretical timer count values of multiples of $T_{1}$ for the standard baud rates and their dependence. In red, the duplicated counter values.

Baud Rate	${1\cdot T}_{1}$	${1.5\cdot T}_{1}$	${2\cdot T}_{1}$	$2.5\cdot T_{1}$	$3\cdot T_{1}$	$3.5\cdot T_{1}$
**2400**	66,666.67	100,000.005	133,333.34	166,666.675	200,000.01	233,333.345
**4800**	33,333.33	49,999.995	66,666.66	83,333.325	99,999.99	116,666.655
**7200**	22,222.22	33,333.33	44,444.44	55,555.55	66,666.66	77,777.77
**9600**	16,666.67	25,000.005	33,333.34	41,666.675	50,000.01	58,333.345
**14,400**	11,111.11	16,666.665	22,222.22	27,777.775	33,333.33	38,888.885
**19,200**	8333.33	12,499.995	16,666.66	20,833.325	24,999.99	29,166.655
**28,800**	5555.56	8333.34	11,111.12	13,888.9	16,666.68	19,444.46
**38,400**	4166.67	6250.005	8333.34	10,416.675	12,500.01	14,583.345
**57,600**	2777.78	4166.67	5555.56	6944.45	8333.34	9722.23
**76,800**	2083.33	3124.995	4166.66	5208.325	6249.99	7291.655
**115,200**	1388.89	2083.335	2777.78	3472.225	4166.67	4861.115
**125,000**	1280	1920	2560	3200	3840	4480
**230,400**	694.44	1041.66	1388.88	1736.1	2083.32	2430.54
**250,000**	640	960	1280	1600	1920	2240
**460,800**	347.22	520.83	694.44	868.05	1041.66	1215.27
**500,000**	320	480	640	800	960	1120
**576,000**	277.78	416.67	555.56	694.45	833.34	972.23
**750,000**	213.33	319.995	426.66	533.325	639.99	746.655
**921,600**	173.61	260.415	347.22	434.025	520.83	607.635
**1,000,000**	160	240	320	400	480	560

**Table 10:** Success rate of interface detection and redirection over 20 repeated connection and disconnection trials for each of the four supported interfaces.

Interface	Connection Procedure		Disconnection Procedure	
	Success (%)	Average Time ± Std. Deviation (ms)	Success (%)	Average Time ± Std. Deviation (ms)
RS-485	100	1277 ± 95	100	2380 ± 257
RS-422	100	1243 ± 55	100	2401 ± 140
RS-232	100	1286 ± 144	100	3048 ± 613
CAN	100	1278 ± 128	100	2154 ± 337

## Data Availability

The source code and the complete electronics design presented in this work are available on request from the corresponding author due to privacy restrictions. Data related to the experiments are included in the Supplementary Materials.

## Supplementary Materials

The following supporting information can be downloaded at: https://www.mdpi.com/article/10.3390/s25247638/s1, File S1 and S2: MATLAB script for serial data raw transmission; File S3: predefined set of data used for transmission; File S4: Complete interface detection and redirection results; File S5: Complete baud rate identification results.

## Abbreviations

The following abbreviations are used in this manuscript:

CAN	Controller Area Network
PCIe	Peripheral Component Interconnect Express
RS	Recommended Standard
PnP	Plug-and-Play
TIA	Telecommunications Industry Association
ASCII	American Standard Code for Information Interchange
LSB	Least Significant Bit
MSB	Most Significant Bit
TTL	Transistor–Transistor Logic
EMC	Electromagnetic Compatibility
IEC	International Electrotechnical Commission
ESD	Electrostatic Discharge
EFT	Electrical Fast Transient
PUL	Pull-up/down Resistors
TVS	Transient Voltage Suppressor
TBU	Transient Blocking Unit
TISP	Totally Integrated Surge Protection
PTC	Positive Temperature Coefficient Fuse
DSP	Digital Signal Processor
ADC	Analog-to-Digital Converter
MCU	Microcontroller Unit
IC	Input Capture
GPIO	General-Purpose Input/Output
I^2^C	Inter-Integrated Circuit
LDO	Low Dropout Regulator
ISR	Interrupt Service Request
EXTI	External Interrupt
